# Epidemiologic study of myelodysplastic syndromes in a multiethnic, inner city cohort

**DOI:** 10.1186/2162-3619-3-22

**Published:** 2014-08-23

**Authors:** Ashwin Sridharan, Rishi Jain, Marcus A Bachhuber, Yiting Yu, KH Ramesh, Krishna Gundabolu, Ellen W Friedman, Amit K Verma

**Affiliations:** 1 Department of Medicine, Albert Einstein College of Medicine, Bronx, NY, USA; 2 Philadelphia Veterans Affairs Medical Center, Philadelphia, PA, USA; 3 Robert Wood Johnson Foundation Clinical Scholars Program, Univeristy of Pennsylvania, Philadelphia, PA, USA; 4 Leonard Davis Insititute of Health Economics, University of Pennsylvania, Philadelphia, PA, USA; 5 Division of Oncology, Albert Einstein College of Medicine, Bronx, NY, USA; 6 Department of Epidemiology & Population Health, Albert Einstein College of Medicine, 302B Chanin, 1300 Morris Park Ave, Bronx, NY 10461, USA; 7 Department of Pathology, Albert Einstein College of Medicine, Bronx, NY, USA; 8 Department of Hematology, Albert Einstein College of Medicine, Bronx, NY, USA

**Keywords:** Myelodysplastic syndrome, Ethnicity, Prognosis, IPSS, IPSS-R

## Abstract

Little is known about the epidemiology of MDS in minority populations. The IPSS and newly released IPSS-R are important clinical tools in prognostication of patients with MDS. Therefore, we conducted a retrospective epidemiological analysis of MDS in an ethnically diverse cohort of patients. Demographics, disease characteristics, and survival were determined in 161 patients seen at Montefiore Medical Center from 1997 to 2011. We observed that Hispanics presented at a younger age than blacks and whites (68 vs. 73.7 vs. 75.6 years); this difference was significant (p = 0.01). A trend towards greater prevalence of thrombocytopenia in Hispanics was observed, but this was not significant (p = 0.08). No other differences between the groups were observed. Overall median survival after diagnosis was the highest among Hispanics (8.6 years) followed by blacks (6.2 years) and Caucasians (3.7). Adjusted hazard ratios however did not show significant differences in risk of death between the groups. The IPSS-R showed slightly better discrimination when compared to the IPSS in this cohort (Somers Dxy 0.39 vs. 0.35, respectively) but observed survival more was more closely approximated by IPSS than by IPSS-R. Our study highlights the possibility of ethnic differences in the presentation of MDS and raises questions regarding which prognostic system is more predictive in this population.

## Introduction

The myelodysplastic syndromes (MDS) are a diverse group of hematological diseases characterized by dysplasia of myeloid lineage cells, peripheral cytopenias, and an increased risk of evolution to acute myeloid leukemia (AML) [1]. Prognosis is highly variable, and a number of scoring systems exist to risk stratify patients and guide decisions on treatment [2-5].

The impact of race on MDS is poorly understood [6]. In the United States, Surveillance Epidemiology and End Results (SEER) data suggests that MDS is more common in whites than in blacks, but survival is equal [7,8]. Retrospective data from the University of Maryland Cancer Center showed slower rates of referral among blacks with MDS versus whites to tertiary care centers, suggesting that there are racial disparities in the care of MDS [9]. Interestingly, this disparity did not lead to differences in outcomes. In a multicenter retrospective trial evaluating adults and children with leukemia and MDS referred for umbilical cord blood transplantation, blacks had inferior survival compared with whites and Hispanics, however, this was suspected to be secondary to difficulty in finding good matches for the black patients rather than inherent characteristics of the disease [10]. A comparison of a cohort of German patients with Japanese patients showed significant differences in incidences of French-American-British (FAB) classifications of MDS; Japanese patients had higher rates of myelodysplastic syndrome unclassified (MDS-U) with pancytopenia and refractory cytopenia with unilineage dysplasia (RCUD) [11,12]. In addition, the Japanese patients had longer overall survival and leukemia free survival compared with the German patients. This study further showed that pathologists in Germany and Japan interpreted the same samples in similar fashions, showing these differences were not due to varying interpretations. A reported cohort from Brazil showed that women comprised a greater percentage of cases than men, a finding contrary to most other countries [13]. The authors suspect that this is because women handle more of the pesticide in that country, implicating environmental factors as a reason for international variability. Furthermore, MDS seems to be diagnosed at different ages in different countries; the results of two studies on small cohorts of patients shows that the median age of diagnosis in Korea, Japan, and German were 57 years, 60 years, and 74 years, respectively [14].

A variety of prognostic systems exist to risk stratify patients with MDS. The Myelodysplastic Syndrome International Prognostic Scoring System (IPSS) has been widely used since being introduced in 1997 [3]. It stratified patients into different risk categories based on percentage of bone marrow blasts, number of peripheral cytopenias, and karyotype and has been used to guide management decisions. In 2012, the IPSS was revised (IPSS-R) [4]. The IPSS-R increased the number of risk categories, expanded the number of cytogenetic abnormalities used, and changed the way scores were computed. Compared to the IPSS, the IPSS-R showed good prediction in a larger cohort of patients. The ethnicity breakdown of the overall cohort has not been published or widely circulated.

Despite the increased awareness of racial and geographic differences in the morphology and prognosis of MDS, little data exists which compares the characteristics of MDS in Hispanics with those of blacks and whites who live in the same geographic area. We conducted a retrospective chart review of patients diagnosed with MDS at a single center, diverse, urban medical center to determine if differences in presentation existed between the three racial groups. Furthermore, we risk stratified all patients according to the IPSS and the IPSS-R to determine if these scores accurately predicted prognosis in our racially diverse cohort.

## Materials and methods

We retrospectively analyzed the charts of all patients diagnosed with MDS at a single center (Montefiore Medical Center) in the Bronx, New York between the years of 1997 and 2011. Using the Clinical Looking Glass software, we identified all patients who presented to an associated hospital or outpatient clinic with an International Classification of Diseases, Book 9 (ICD-9) diagnosis or related diagnosis of MDS. Individual codes used were 238.72, 238.73, 238.74, and 238.75, which correspond with diagnoses of “Low grade MDS lesions,” “High grade MDS lesions, “MDS with 5Q deletion,” and “MDS, unspecified,” respectively. Clinical Looking Glass is a software tool which allows for the collection and analysis of patient data (e.g., demographic and laboratory data) for all visits to our institution between the years of 1997 and the present. Patient records containing an ICD-9 diagnosis code for MDS were further examined for bone marrow biopsy results; only patients’ bone marrow biopsy results consistent with MDS by World Health Organization (WHO) criteria were included into the study. Patients with treatment-related MDS were not excluded.

Demographic information (age, sex, and ethnicity) was obtained from the medical record. Information regarding ethnicity was collected through patient self-report questionnaires. Ethnic categories were “White”, “Black”, “Hispanic”, “Other”, and “Unknown”. Hispanics could be of any race. Few patients reported “Other” (n = 1) and “Unknown” (n = 2) race/ethnicity and we did not include them in our analyses.

Laboratory data (peripheral blood counts and bone marrows biopsy results including cytogenetic findings) were also recorded from the medical record. Anemia was defined as hemoglobin < 10 g/dL, thrombocytopenia as < 100,000 platelets per microliter, and neutropenia as < 1,800 neutrophils per microliter. Cytogenetics were determined by metaphase cytogenetics and fluorescent in-situ hybridization (FISH) with a panel of DNA probes for clinically relevant derangements. Probes for 5q31, monosomy 5, monosomy 7, deletion 7q31, trisomy 8, TP53, and deletion 20q12 were routinely checked for by FISH. For each patient, IPSS and IPSS-R risk category were calculated according to published methods [3,4].

We defined the date of MDS diagnosis as the date of the first bone marrow biopsy consistent with MDS in our record. To determine the date of death, we examined the medical record and the Social Security Death Index. If the patient died at our institution, we recorded the date of death from the medical record. For patients without a date of death in the medical record, we queried the Social Security Death Index. If no date of death was found in the Social Security Death Index query, we censored the patient on the day the query was performed. The study protocol was approved by the Montefiore Medical Center Institutional Review Board.

### Statistical analysis

To compare differences in baseline presenting characteristics between Black, White, and Hispanic patients with MDS, we compared continuous variables (e.g., age) using the Kruskall-Wallis test and categorical variables (e.g., race/ethnicity, presence of peripheral cytopenias, IPSS and IPSS-R risk group) using the chi-square or Fisher exact test, where appropriate. No adjustments for multiple testing were applied.

Next, to examine survival of Black, White, and Hispanic patients with MDS, we constructed Kaplan-Meier curves and compared unadjusted survival using the log-rank test. Then, we compared survival between Black, White, and Hispanic patients with MDS after adjusting for demographics and IPSS/IPSS-R risk group using multivariable Cox proportional hazards regression models. We developed three separate multivariable models: 1) adjusting for age and sex; 2) adjusting for age, sex, and IPSS risk score; and 3) adjusting for age, sex, and IPSS-R. In each model, we verified the proportionality assumption for all independent variables.

Finally, we examined the performance of the IPSS and IPSS-R in our cohort of patients. To assess discrimination, the ability of the IPSS or IPSS-R score to distinguish between high risk and low risk, we calculated Somers’ Dxy for right-censored survival data [15]. To assess calibration, the degree to which the observed survival in our cohort was similar to the survival predicted by IPSS or IPSS-R, we constructed graphs of observed versus predicted survival. Statistical analyses were performed with SAS 9.3 (SAS Institute, Cary, NC, USA) and R 3.0 (R Foundation for Statistical Computing, Vienna, Austria); a two-tailed P-value ≤ 0.05 was considered significant.

## Results

**Figure 1:**
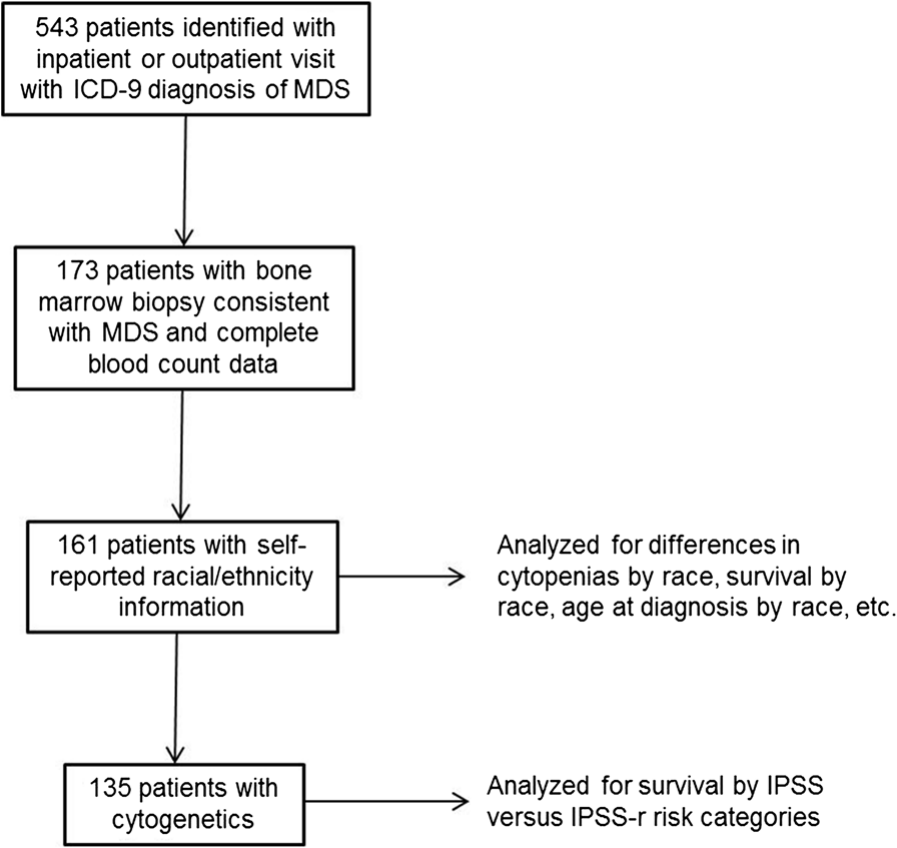
**Summary of patients included in analysis.** ICD-9 = International Classification of Diseases, Book 9, IPSS-R = Revised international prognostic scoring system, IPSS = International prognostic scoring system.

A total of 543 patients were identified who visited Montefiore outpatient or inpatient facilities with an ICD-9 diagnosis of MDS between the years of 1997 to 2011(Figure 1). Of these patients, 173 had bone marrow biopsies that were consistent with MDS and were included in the analysis. The remaining patients either did not have bone marrow biopsies in our system, had bone marrow biopsies that were normal, or had diagnoses consistent with other hematological disorders (including lymphoma, multiple myeloma, de novo acute myeloid leukemia, etc.). Complete demographic and laboratory data were available for 161 of these patients. Results of cytogenetic studies were available in 84% (135/161) of patients, which allowed computation of IPSS and IPSS-R risk categories.

### Analysis of patients with complete demographic and laboratory data

**Figure 2:**
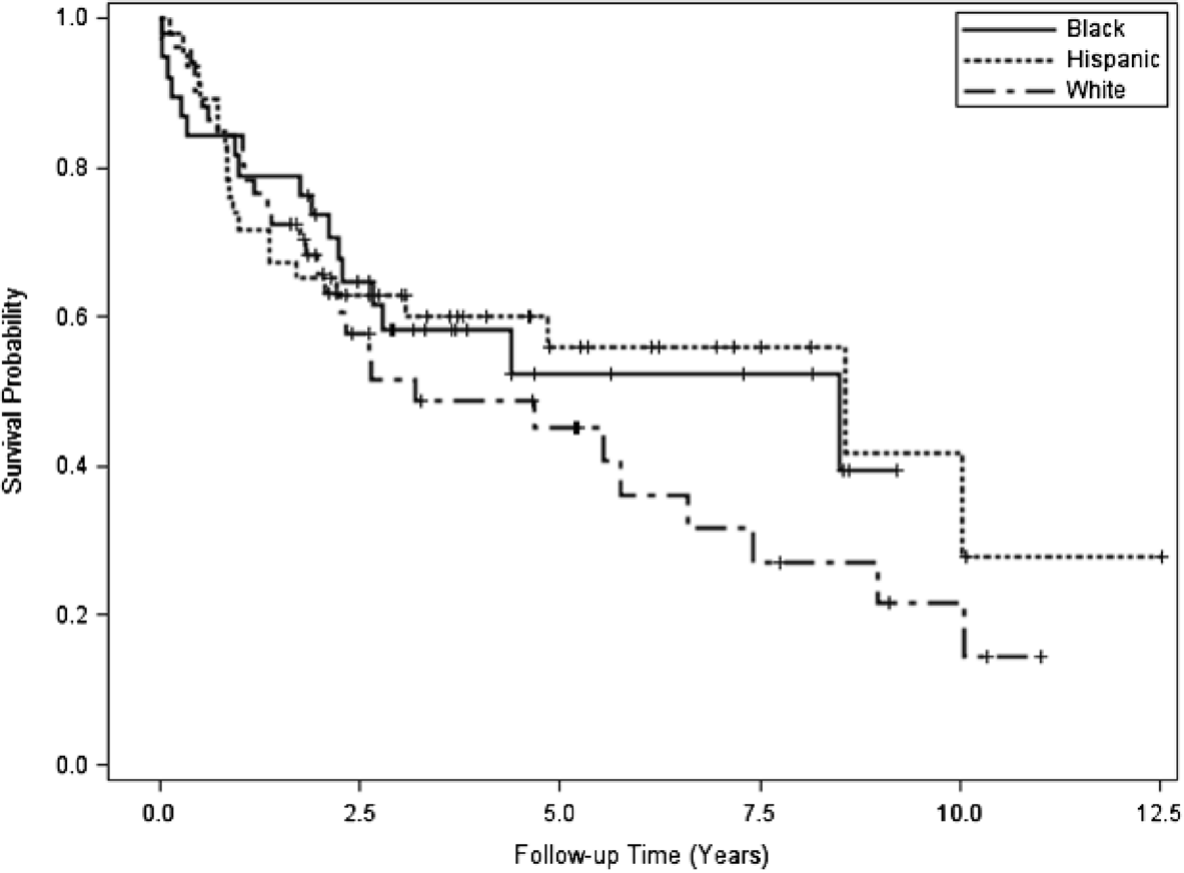
Kaplan-Meier survival curves by ethnicity.

Of patients with complete demographic and laboratory data (n = 161), the median age was 72.5 (IQR: 14.9) with 50.3% (n = 81) women and 49.7% (n = 80) men (Table 1). By race, 34% (n = 54) were Black, 30% (n = 49) were Hispanic, and 36% (n = 58) were White. Age at diagnosis was significantly different between the racial/ethnic groups (P = 0.01). A trend towards increased prevalence of thrombocytopenia was observed in Hispanics, however, these results were not significant. No other differences in prevalence of cytopenias were noted between the groups (Table 2). During 590.2 person-years of follow-up, 52.2% (n = 84) patients died; median survival was 5.8 years after diagnosis. Unadjusted survival was not significantly different between Black (6.2 years), White (3.7), and Hispanic patients (8.6 years; P = 0.28; Figure 2).

**Table 1:** Demographic characteristics at diagnosis for all patients persons by ethnicity

**Characteristic**	**All (n = 161)**	**Black (n = 54)**	**Hispanic (n = 49)**	**White (n = 58)**	**p-value**
Age, y					
Median (IQR)	72.5 (14.9)	73.7 (16.1)	68.0 (21.7)	75.6 (14.0)	0.01
Sex, n (%)					
Female	81 (50.3)	29 (53.7)	28 (57.1)	24 (41.4)	0.22

n = number of patients, IQR = interquartile range.

**Table 2:** Frequency of cytopenias for all persons by ethnicity

**Cytopenia, n (%)**	**All (n = 161)**	**Black (n = 49)**	**Hispanic (n = 49)**	**White (n = 58)**	**p-value**
Anemia	92 (57.1)	30 (55.6)	29 (59.2)	33 (56.9)	0.93
Thrombocytopenia	70 (43.4)	18 (33.3)	27 (55.1)	25 (43.1)	0.08
Neutropenia	18 (11.1)	5 (9.3)	4 (8.2)	9 (15.5)	0.49

n = number of patients.

### Analysis of patients with complete cytogenetics data

Among the patients with results of bone marrow cytogenetic studies (n = 135), no significant differences in the distribution of cytogenetic abnormalities by ethnicity or prevalence of risk categories by ethnicity were noted (Table 3). During 458.9 person-years of follow-up, 50.4% (n = 68) patients died; median survival was 5.5 years after diagnosis and survival was not significantly different between Black, White, and Hispanic patients in unadjusted (P = 0.51) and adjusted analyses (Table 4).

**Table 3:** Myelodysplastic syndrome risk score for all persons by ethnicity

**Risk score, n (%)**	**All (n = 135)**	**Black (n = 38)**	**Hispanic (n = 46)**	**White (n = 51)**	**p-value**
IPSS					0.62
Low	65 (48.1)	20 (52.6)	20 (43.5)	25 (49.0)	
INT-1	46 (34.1)	15 (39.5)	15 (32.6)	16 (31.4)	
INT-2	21 (15.6)	3 (7.9)	9 (19.6)	9 (17.6)	
High	3 (2.2)	0 (0)	2 (4.3)	1 (2)	
IPSS-R					0.33
Very Low	27 (20)	9 (23.7)	7 (15.2)	11 (21.6)	
Low	54 (40)	18 (47.4)	18 (39.1)	18 (35.3)	
Intermediate	27 (20)	5 (13.2)	11 (23.9)	11 (21.6)	
High	10 (7.4)	4 (10.5)	1 (2.2)	5 (9.8)	
Very High	17 (12.6)	2 (5.2)	9 (19.6)	6 (11.7)	

n = number of patients, IPSS = International prognostic scoring system, IPSS-R = Revised international prognostic scoring system.

**Table 4:** Adjusted hazard ratios for mortality by ethnicity

**Ethnicity**	**Adjusted for age, sex**	**Adjusted for age, sex and IPSS**	**Adjusted for age, sex and IPSS**-**R**
	**HR (95% CI)**	**HR (95% CI)**	**HR (95% CI)**
Black	Reference	Reference	Reference
White	1.28 (0.70-2.40)	0.92 (0.49-1.78)	0.87 (0.46-1.171)
Hispanic	0.90 (0.47-1.74)	0.72 (0.37-1.42)	0.67 (0.33-1.35)

HR = hazard ratio, CI = confidence interval, IPSS = International prognostic scoring system, IPSS-R = Revised international prognostic scoring system.

**Figure 3:**
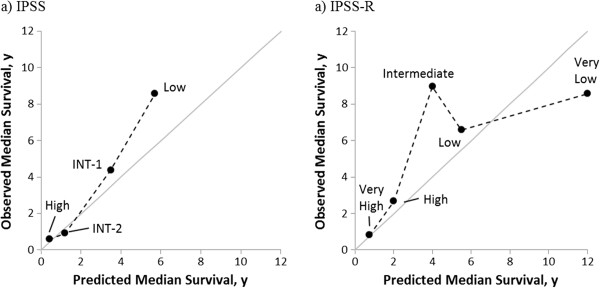
**Observed vs. predicted survival by A) IPSS and B) IPSS-R.** IPSS = International prognostic scoring system, IPSS-R = Revised International prognostic scoring system, y = years.

Next, median survival was determined for according to IPSS and IPSS-R risk categories (Table 5). Discrimination of IPSS-R (Somers’ Dxy = 0.39) was slightly better than IPSS (Somers’ Dxy = 0.35). Observed median survival more closely approximated predicted survival with IPSS than with IPSS-R (Figure 3). For IPSS-R, patients classified as “Intermediate” risk had a longer median survival (9.0 years) than patients classified as “Low” (6.6 years) and “Very Low” risk (8.6 years).

**Table 5:** **Estimated median survival by IPSS and IPSS**-**R categories**

**IPSS**	**Survival, observed (y)**	**Survival, reported (y)**	**IPSS-R category**	**Survival, observed (y)**	**Survival, reported (y)**
Low	8.6	5.7	Very Low	8.6	8.8
INT-1	4.4	3.5	Low	6.7	5.3
INT-2	0.9	1.2	Intermediate	9.0	3.0
High	0.6	0.4	High	2.7	1.6
			Very High	0.8	0.8

IPSS = International prognostic scoring system, IPSS-R = Revised international prognostic scoring system, y = years.

## Discussion

Our analysis for the first time examines the impact of race on the epidemiology of MDS within a single center. Previous studies have focused on the differences between WHO morphology from different centers; specifically, between Asian and European ones. In our cohort, Hispanics were diagnosed at a younger age when compared with non-Hispanic blacks and whites. Part of this effect may be related to the demographics of the Bronx; the Hispanic population tends to be younger than the non-Hispanic black and white population. Additional data from other centers would be useful in assessing if this observation is limited to this center or if in fact Hispanics do develop MDS at a younger age. Furthermore, prevalence of thrombocytopenia in Hispanics seemed to be increased but was not statistically significant. Neither of these findings resulted in differences in survival between the ethnic groups, and there were no differences between distributions by risk score for the different ethnicities.

The more surprising portion of our analysis was the observation that the IPSS more accurately predicted survival than the IPSS-R. This was mediated mostly by better than expected survival in “Intermediate” IPSS-R group. We had insufficient number of patients to compute meaningful confidence intervals, which raises the question that this finding was a statistical anomaly. It is possible that ethnicity may impact risk stratification in MDS, but how is not clear. Ethnic cohorts were overall similar to each other based on clinical criteria used to stratify patients by IPSS and IPSS-R, and distribution by risk score was similar as well. A comparison of Somer’s dxy’s by different ethnic groups could help determine if the IPSS or IPSS-R performs better for different ethnic groups; unfortunately, our patient numbers were too small to perform such an analysis. Larger studies comparing performance of these systems in multi-ethnic populations may help answer this question.

Other single center studies have shown that the results of the IPSS-R, while predictive on a large scale, may not be predictive in smaller, local cohorts. An analysis of 783 patients from Mayo Clinic showed that IPSS-R karyotype categories failed to discriminate between better and worse prognoses; for example, patients in the “very good” cateogory had a median survival of 21 months compared with median survivals of 40 and 24 months for patients with “good” and “intermediate” cytogenetics, respectively [16]. The authors found that patients with monosomal karyotypes had worse survival and suggested including these patient’s into the “very poor” category.

Our study does have several limitations. MDS as a disease is often prone to referral bias, as more indolent forms of the disease are less likely to be referred for bone marrow biopsy. However, a significant portion of the identified cohort was noted to have lower grade disease. How this bias has affected these results remains unclear. Furthermore, we were unable to access data regarding the treatment which these patients received, and this information could not be included in our analysis. As a retrospective analysis, we were reliant on accurate identification of patients’ medical records through ICD-9 codes. We are unsure if ICD-9 codes changed between the years of 1997 to 2011, but this may have introduced the possibility of not identifying the records of some patients with MDS. Furthermore, inaccurate ICD-9 coding could have limited our ability to identify a larger cohort of patients.

All patients were drawn from a single center, which limits the generalizability of these results. Although differences were observed in survival by ethnicity, our sample size was too small to say these were not the result of chance alone. The SEER database, which includes information on tumor type, ethnicity, and survival, would likely be more definitive in answering questions regarding differences in survival by ethnicity. Finally, although some observed trends reached statistical significance, our sample size was small which may have limited our ability to detect other differences that actually might exist. Further analysis in large, prospective, multi-center cohorts may help better answer whether race has an impact in the presentation of prognosis of MDS.

## Competing interests

The authors declare that they have no competing interests.

## Authors’ contributions

AS wrote the manuscript, was involved in study design, and conducted chart review. RJ was involved in study design and conducted chart review. MB was involved in study design, performed the statistical analysis, and edited the manuscript. All authors read and approved the final manuscript. YY also aided in the statistical analysis. KHR supplied the cytogenetics data. KG archived patients treated with at our institution and recorded and performed cytogenetics. EWF edited the manuscript and aided in interpretation of the results. AV was involved in study design, interpretation of the results, and edited the manuscript.
